# Molecular characterization of two distinct monopartite begomoviruses infecting tomato in india

**DOI:** 10.1186/1743-422X-7-337

**Published:** 2010-11-23

**Authors:** Prerna Pandey, Subhra Mukhopadhya, Afsar R Naqvi, Sunil K Mukherjee, Gyan S Shekhawat, Nirupam R Choudhury

**Affiliations:** 1 Department of Bioscience and Biotechnology, Banasthali University, Banasthali-304020, Rajasthan, India; 2 Plant Molecular Biology Group, International Centre for Genetic Engineering and Biotechnology (ICGEB), Aruna Asaf Ali Marg, New Delhi-110 067, India; 3 Department of Biosciences, Jamia Millia Islamia, New Delhi-110025, India

## Abstract

**Background:**

*Tomato leaf curl viruses*, which are the members of the genus *Begomovirus*, have emerged as devastating pathogens worldwide causing huge economic losses and threatening production of crops like cassava, cotton, grain legumes and vegetables. Even though the ToLCV isolates from Northern India have been shown to possess bipartite genome (designated as DNA A and DNA B), those from Australia, Taiwan and Southern India have a single genomic component (DNA A). We describe here the genetic diversity of two isolates of monopartite *Tomato leaf curl virus *infecting tomato in two extreme regions (North and South) of Indian subcontinent.

**Results:**

The rolling circle amplification (RCA) products obtained from symptomatic samples were digested, cloned and sequenced. The complete DNA sequence of two *Tomato leaf curl virus *isolates identified as ToLCV-CTM (India, New Delhi, 2005) and ToLCVK3/K5 (India, Kerala, 2008) are reported here. These isolates had the characteristic features of Begomovirus genome organization with six conserved open reading frames (ORFs). The ToLCV-K3 and ToLCV-K5 isolates may be the strains of the same virus since they show sequence homology of 97% over their entire genome. This, according to the guidelines established by the ICTV *Geminiviridae *Study-Group is higher than threshold (92%) for delineation of different viral variants and hence single, average value has been assigned for all their analyses presented here. The ToLCV-CTM and ToLCV-K3/K5 viruses were found to be monopartite, as neither DNA-B component nor betasatellite associated with begomovirus species, were detected. The complete nucleotide sequence of DNA-A genome of CTM exhibited highest sequence homology (88%) to *Croton yellow vein mosaic virus *(AJ507777), and of isolates K3/K5 (88.5%) to *Tomato leaf curl Pakistan virus *(DQ116884). This is less than the threshold value for demarcation of species in the genus *Begomovirus*.

**Conclusion:**

K3/K5 and CTM are considered to be novel isolates of *Tomato leaf curl virus*. Sequence analyses and phylogenetic study indicate that these two ToLCV isolates might have evolved by recombination between viruses related to two or more viral ancestors. The existence of different ToLCV isolates having high genome diversity in India poses a threat to the tomato production in the Asian continent.

## Background

Geminiviruses are plant viruses characterized by twin icosahedral particles [1]. These viruses are divided into four genera, viz., *Mastrevirus*, *Curtovirus*, *Topocuvirus *and *Begomovirus*, on the basis of the viral vector, genome organization and host range. Geminiviruses transmitted by whitefly belong to the genus *Begomovirus*, have either bipartite genome (known as DNA-A and DNA-B, both being ssDNA genomes of approximately 2.7 kb size, and contain ~ 220 bp Common Region) or a monopartite genome [2,3]. Monopartite genomes are homologous to the DNA-A of bipartite geminivirus, and all the viral factors required for viral replication, encapsidation, transmission, and systemic spread are encoded on this genome component [2]. Monopartite begomoviruses have also been associated with a novel DNA component, the DNA β or betasatellite, which is approximately half the size of the helper virus genomic DNA [4]. Studies have demonstrated that the cloned monopartite begomoviruses, when inoculated to the susceptible host, give rise to disease symptoms like those of the field isolates [5].

*Tomato leaf curl virus *(ToLCV) is a member of the family Geminiviridae (*Begomovirus *genus) and are more common in tropical and subtropical climates [6,7]. It is a major causative agent for reduction in tomato production in different parts of the world and may also infect other *Solanaceous *species. The affected plants are severely stunted, leaflets become reduced in size, pucker, curl upwards, become distorted and have prominent yellow margins. The infection severely affects healthy fruit formation if it sets in when the plant is young. The virus is transmitted by whiteflies (*Bemisia tabaci*) that are attracted to young leaves and growing tips.

The discovery of ToLCV in the Indian subcontinent together with its spread has aggravated the ToLCV situation. In such context the study of the identity, distribution, molecular variability, and the threat that these emerging geminiviruses pose to tomato production in India and more generally in Asia and Africa, has become very important. The recombination between two species of geminiviruses was first recorded in 1996 [8,9]. This mechanism is now known to be widely prevalent in all geminiviruses and is probably the most important molecular mechanism for generating genetic changes that allow novel geminiviruses to exploit new ecological niches [10]. This paper describes the results of a molecular study of the sequences of genomes of different ToLCV isolates collected from the Indian subcontinent in an effort towards identifying and determining the molecular variability of ToLCVs.

In view of evolution of variants of Begomovirus family of plant viruses, it becomes imperative to characterize the diseased plants for the identification of isolates causing infection. Recently, the identification and isolation of geminiviral genome has become much easier by the use of Phi-29 polymerase, a DNA polymerase that exponentially amplifies circular DNA templates by rolling circle amplification (RCA), using picogram amounts of the starting material [11]. Phi-29 polymerase also possesses proofreading activity and thus ensures high fidelity DNA replication.

In this study, we describe the molecular characterization of two new monopartite *Tomato leaf curl virus *isolated from symptomatic tomato plants growing in two different regions of India. Although the symptoms were severe, we could not detect either DNA-B or betasatellite associated with these new begomovirus species. A phylogenetic relationship of these isolates was established with other previously characterized begomoviruses, indicating towards the fact that genetic recombination has probably featured prominently in the evolution of these viruses.

## Results

### Identification, detection and molecular characterization of novel monopartite begomoviruses

**Figure 1:**
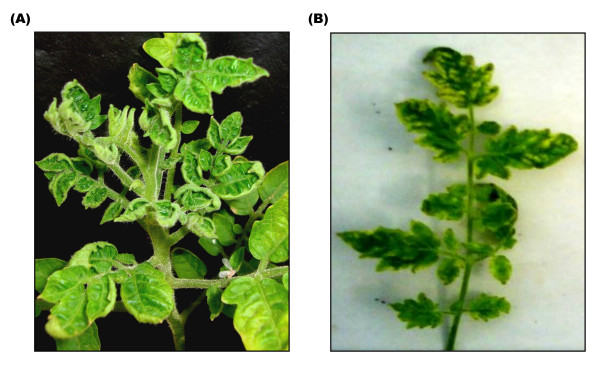
**Differences in the phenotype of infected leaves of tomato cv Pusa Ruby. **(A) Leaves field infected with ToLCV-CTM isolate showing upward leaf curling. (B) Glasshouse infection mediated by ToLCNDV (2A+2B) genomes lead to the severe mottling and yellowing of the leaves.

**Figure 2:**
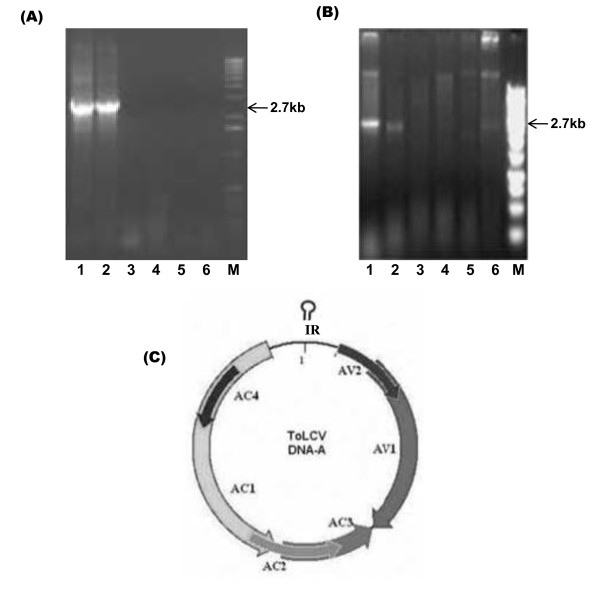
**Detection of viral genome in infected tomato leaf samples and the ORFs encoded by Tomato leaf curl virus**. (A) Rolling Circle Amplification (RCA) of total DNA for ToLCV-K3/K5 samples using Ø29 polymerase digested by *Bam H*I (lanes 1, 2), *Xba *I (lanes 3, 4) and *Xho *I (lanes 5, 6). (B) RCA of total DNA for ToLCV-CTM digested by *Bam H*I, *EcoR *I, *Sma *I, *Nco *I, *Xmn *I and *Nde *I (lanes 1-6 respectively). M denotes size marker lane. (**C**) Genome map of Tomato leaf curl virus DNA A showing the sense (AV1and AV2) and complementary strand ORFs (AC1, AC2, AC3, AC4).

**Figure 3:**
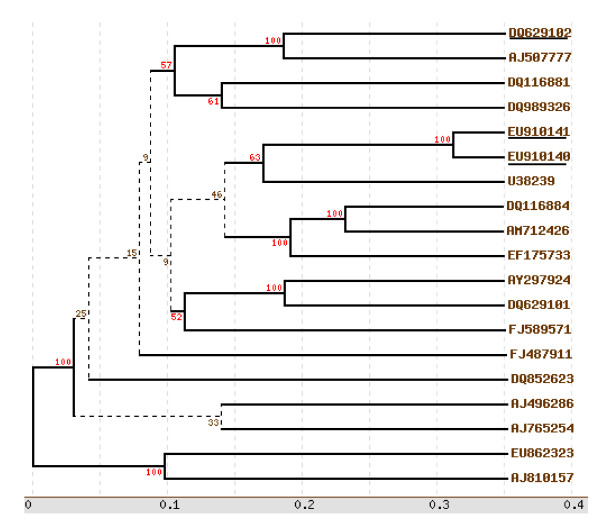
**Phylogenetic relationship of complete genome of the CTM, K3/K5 isolates  with other Old World Viruses.** The phylogenetic tree was constructed on the basis of complete genome sequence. Trees were prepared using Treeview programs and are based on 100 bootstrapped data sets. All the sequences used in this analysis were collected from GenBank. The database accession numbers for each isolate are mentioned in the text (Table 1). The new isolates mentioned in this study are underlined.

Leaves of infected tomato plants were collected from two different regions of Indian subcontinent and checked for the presence of the causal agent. The field infected tomato cv Pusa ruby leaves showing upward leaf curling (Figure 1A). On the other hand, tomato plants agroinfected with both ToLCNDV 2A and 2B genomes exhibit severe infection with symptoms of leaf mottling and yellowing (Figure 1B). Complete nucleotide sequences of the full length (~2.7 kb) genomes of the two isolates amplified by the RCA technique were found to be monopartite. The genomes are 2751-2769 nucleotides in length and are referred to as DNA A since they show maximum homology to the DNA A of previously characterized begomoviruses. The viruses were named as ToLCV-CTM and ToLCV-K3/K5 and the sequences are submitted to NCBI under accession numbers DQ629102 and EU910141/EU910140 respectively. We could not detect the presence of any second genomic component (betasatellite or DNA-B), as revealed by PCR (using the betasatellite DNA specific primers) or the RCA by the phi-29 polymerase technique (Figure 2A, B). Each of the DNA A sequences contained four complementary sense ORFs (AC1, AC2, AC3 and AC4) and two virion sense ORFs (AV1, AV2) and has been represented as a common genome map of the virus (Figure 2C). The degree of relationship of the nucleotide sequence of Intergenic Region (IR) and the amino acid sequences of the proteins expressed by the viral genomes of the three ToLCV isolates is illustrated in Table 1. Sequence comparisons of the three genomes and the phylogenetic analysis (Figure 3) showed that they were more closely related to Old world viruses.

**Table 1:** Homology analysis of the three ToLCVs with other geminiviruses.

Accession	Virus species/virus isolates	Identity- species	IR - nt	AC1-aa	AC2-aa	AC3-aa	AC4-aa	AV1-aa	AV2-aa
DQ629101	Tomato leaf curl virus isolate ToLCND-CTS	83/80	68/78	82/72	89/87	79/82	46/22	79/80	80/84
**EU910140/** **EU910141**	Tomato leaf curl Kerala virus isolate ToLCV-**K5/K3**	97/83.5	100/64	92/69	91/87.5	99/88	93/22	97/93	100/91
**DQ629102**	Tomato leaf curl virus isolate ToLCND-**CTM**	83.5/100	64/100	69/100	87.5/100	88/100	22/100	93/100	91/100
DQ116884	Tomato leaf curl virus isolate Rahim Yaar Khan, PT7	88.5/83	82/65	92/71	91/86	88/88	89.5/22	95/94	89/88
AJ507777	Croton yellow vein mosaic virus	85/88	55.5/64	72/86	88/97	92/89	43/57	81/82	85/89
U38239	Tomato leaf curl virus - Bangalore II	87/87	67/65	81/71	93/87	91/91	46/27	94/96	92/96
AY297924	Tomato leaf curl virus -	82/81	63/77	78/68	95/88	92/89	42/21	79/81	86/89
DQ852623	Tomato leaf curl virus - [Kerala II 2005]	79.5/78	39/47	79.5/69	84/80	86/87	41/23	88.5/89	73/71
EU862323	Tomato leaf curl virus	81/75	51/57	80/66	66/63	67/63	87/24	82/80	83/80
AJ496286	Cotton leaf curl Kokhran virus	85.5/82	83/55	83/71	90/85	85/85	62/32	94/95	91/96
AY765254	Cotton leaf curl Rajasthan virus	81/75	71/45	81/67	67/66	70/70	62/32	92/92	72/71
AM712436	Pedilanthus leaf curl virus	86/81	81/55	91/71	91/86	88/88	80/31	94/94	84/86
DQ116881	Pepper leaf curl virus	79.5/85	57/79	71.5/85	86.5/84	79/75	42/51	94.5/94	87/85
DQ989326	Papaya leaf curl virus	81/81	58/58	82.5/74	86.5/84	79/73	50,24	93/94	87/84
EU482411	Bhendi yellow vein mosaic virus	83/71	64/58	74/67	61/61	68/67	44/15	93/92	74/73
EF175733	Radish leaf curl virus	87/82	58/61	91/72	85/82	85/85	84/24	95/95	91/95
FJ487911	Euphorbia leaf curl virus-	83/81	55/71	92/74	91/83	86/83	48/27	91/91	75/78
AJ810157	Stachytarpheta leaf curl virus - [Hn34]	81/73	81/73	89/67	70/65	66/61	84/30	82/80	76/74

Each column (IR, AC1-4, AV1, AV2) shows two values: first value is the percent identity of ToLCVK3/K5 (EU910140/EU910141) with other viruses, and the second value is the percent identity of ToLCV-CTM (DQ629102) with other viruses. The underlined values denote the highest values for any identity analysis

### Sequence analysis of the genomes of new strains of Tomato leaf curl virus and comparison with other begomoviruses

Figure 2 shows a genomic map for each component, while Table 1 summarizes the results obtained for the genomes of both the isolates. ORF-wise amino acid sequence identity comparison revealed that the genomes of the viral isolates under study share highest sequence identity with different begomovirus isolates reported from the Indian subcontinent and thus indicated the possibility of recombination. To test for any possible recombination event, we first constructed a nucleotide sequence alignment of ToLCV with each of the geminivirus genome sequences used in this study. The pairwise as well as RDP3 software analyses performed with the sequences of both the ToLCV Indian isolates indicated the occurrence of multiple overlapping recombination events with different parental combinations. The A components of most of the viruses in South and North India shared a common backbone from *Tomato leaf curl virus*-Bangalore II (ToLCV-BII, Accession number U38239) (Figure 3, See additional file 1) and have integrated other pieces of DNA that have been proposed to originate from the other viruses not identified so far.

### The *Tomato leaf curl virus- New Delhi *(ToLCV-CTM)

As depicted in Table 1, the ToLCV-CTM isolate showed 88% homology with *Croton yellow vein mosaic virus *(CrYVMV-AJ507777), suggesting it to be a novel strain of CrYVMV. The virus isolate exhibited a putative recombination with the viral isolates used in this analysis (See additional file 1), and the result indicates that *Croton yellow vein mosaic virus *is the major parent. The other minor parents appear to be *Tomato leaf curl virus *(U38239, ToLCV-BII; EU910140, ToLCV-K3/K5; DQ852623, ToLCV-KII). In depth analysis identified at least two significant segments for the CTM isolates, where the crossover has occurred with the ToLCV-K5 (EU910140; nt 1091-1137, 2643-2692; spanning the AC3 and IR). The other putative crossover events appear to be with ToLCV-BII (nt 2308-2326; spanning the AC1) and with ToLCV-KII (nt 2434-2510; spanning the AC1). Besides this, there were three unknown recombination sites in the CTM genome. Moreover, the CR IR region also shows an identity of only 65% to ToLCV-BII and 64% to CrYVMV.

### The *Tomato leaf curl virus- Kerala *(ToLCV-K3/K5)

According to the guidelines established by the ICTV *Geminiviridae *Study-Group, two geminivirus sequences sharing more than 89% identity of their A component sequences are considered strains or isolates of the same species. The level of sequence homology (97%) over the entire genome of ToLCV-K3 and ToLCV-K5 isolates was observed to be higher than the threshold (92%) for delineation of different viral variants, suggesting that K3 and K5 may be the different strains of the same virus, and hence a single, average value has been assigned for all the subsequent analyses. The full length genomes of K3/K5 virus showed 88.5% homology with *Tomato leaf curl Pakistan virus *isolate Rahim Yaar Khan (ToLCPV, DQ116884), indicating these to be novel isolates of the genus *Geminiviridae*. The virus isolate exhibited a putative recombination, and interestingly, CrYVMV (AJ507777) appears to be a major parent besides DQ116884, ToLCPV (See additional file 1). The minor parents appear to be isolates of *Tomato leaf curl virus, viz*., U38239 (ToLCV-BII) and DQ852623 (ToLCV-KII). In depth analysis identified that at the probability of 3.26 X10^-74^, U38239 ToLCV-BII could be a major parent and ToLCPV could be a minor parent (nt 24-982, spanning the AV1 and AV2). As seen from the Additional file 1, Table S1, one of the important recombination sites lies in the AC1 region of the genome and the other could be the IR region. Besides, there was one unknown recombination site identified for the ToLCV isolate EU910141. We observed a recombination event with significant probability for AJ507777 and EU910140 indicating that a major portion of EU910140 has been incorporated into AJ507777 during evolution. Moreover, the IR of ToLCV-K3/K5 is homologous by only 82% to that of ToLCPV and 67% to ToLCV-BII.

### Phylogenetic analysis and sequence comparison with selected viruses

The CP gene sequences of the two ToLCVs identified in our study were compared to published sequences (Table 1). The amino acid sequence of CTM V1 ORF was almost identical (96%) to that of ToLCV-BII (Figure 3), while the C1 ORF showed high similarity (86%) to that of CrYVMV. Likewise, the amino acid sequence of K5/K3 was 95% homologous to the V1 ORF of ToLCPV and the C1 ORF exhibited high similarity (95%) to that of ToLCPV and *Euphorbia leaf curl virus *-Fujian (FJ487911). The full length genomes of both the characterized isolates show 77-80% homology to ToLCV- Ban4 (AF165098) another well characterized monopartite *Tomato leaf curl virus *[12, data not shown].

The coat proteins of the isolates under study (K3/K5, CTM) displayed 79% to 96% homology with other ToLCV isolates, while that of K3/K5 showed 95% homology with *Radish leaf curl virus *(EF175733) and contain two basic domains of KR and KVRRR at the N-terminus. The pre-coat protein regions had closest homology (CTM 96% and K3/K5 92%) to that of ToLCV-BII (U38239) as shown in Table1. The CP gene of whitefly transmitted geminiviruses typically end with a double stop codon (TAATAA), the first and the second nucleotides of which are the second and first nucleotides respectively of the stop codon of the complementary C3 gene [13], as observed in the case of ToLCV-CTS, a previously characterized monopartite *Tomato leaf curl virus *(DQ629101; data not shown). However, this overlap is eliminated and there are separate stop codons for CP and C3 in the case of isolates K3/K5. This variation does not lead to any change in the last amino acids (viz., VTN) of the CP of isolates K3/K5; however, there is a change in the last three amino acids of CTM (viz., VSN), that is similar to *Pedilnthus leaf curl virus *(AM712436).

The C2 protein regions had closest homology (CTM 97%) to the C2 of CrYVMV and (K3/K5 95%) to the C2 of ToLCV- Iran (AY297924) as shown in Table1. The alignment of the C2 protein of K3/K5 and CTM with other begomoviruses revealed that, like that of *Tomato yellow leaf curl virus *C2 protein [14], a putative zinc-finger motif C36-X1-C38-X7-C46-X6-H53-X4-H58C59 and four potential phosphorylation sites (T52, S61, Y68, and S74) are present (Figure 2B). The identity of C4 protein of K3/K5 and CTM ranged from 22% to 93%, having highest homology to ToLCPV isolate for K3/K5 (89.5%), and to CrYVMV isolate for CTM (57%).

### The Intergenic Region (IR) sequences

**Figure 4:**
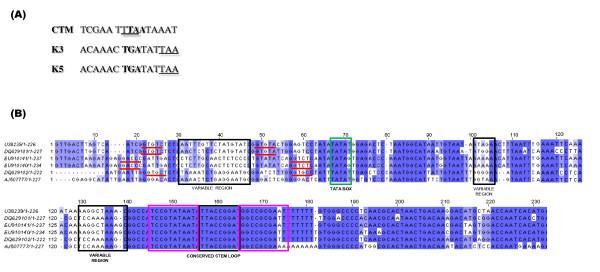
**Sequence analysis of the coat protein and CR of the newly identified ToLCV isolates.** (A) Nucleotide sequence of the 3' region of the CP gene of the two new ToLCV isolates from India (CTM, K3/K5). The sequence of isolate CTM is typical for the whitefly transmitted geminiviruses in having overlapping CP and AC3 termination codons. (B) The alignment of CR sequences of ToLCVs that have highest homologies with the new viral isolates. The iterons for each ToLCV CR sequence are represented in underlined bold red. The TATA box, the conserved regions and stem-loop regions are also indicated in the figure.

A characteristic feature of genome of whitefly transmitted geminiviruses is the Intergenic Region, or, IR where, except for the nonanucleotide sequences, the region is especially prone to variation and provides a sensitive guide to differences between isolates. The IR is known as CR (Common Region) in the case of bipartite begomoviruses or monopartite begomoviruses having betasatellite molecules. When the IR sequence of K3/K5 and CTM isolates was compared and aligned to the published IR sequences of other Old world geminiviruses from Asia (Table 1), it was apparent that the IR from ToLCV- K3/K5 and CTM isolates were highly dissimilar to all other ToLCV isolates and other geminiviruses. There are marked differences in both the sequence and arrangement of Rep-binding sequences (Iterons) of these viruses (GGTGC/GGTGC for the CTM isolate, and GGACC/GGTCT for the K3/K5 isolate) as is shown in Figure 4B. The IR region for the ToLCV-K3 is 100% homologous to the ToLCV-K5 and exhibit 97% homology with each other on the whole genome basis (they could be variants of the same strain since they were isolated at the same time, from the nearby plants). The sequence and arrangement of IR and iterons were identical for the K3/K5 isolates showing 91% homology to Whitefly transmitted Indian begomovirus from *Parthenium hysterop *(DQ339128; data not shown) and differed from that of CTM isolate (only 64% homology; Table 1). The IR of CTM isolate displayed 97% homology with *Tomato leaf curl New Delhi virus *AC1 gene (FN645906; data not shown). The Intergenic Region of all geminiviruses contain stem loop with conserved (in most of the cases) nonanucleotide sequence 'TAATATTAC'. Blocks of conserved sequences can also be seen in Figure 3 at the middle and the 3' regions, but there is a lot of variation in the 5' region to the conserved TATA motif and in the AT rich sequence between the TATA motif and the nonanucleotide sequence (Figure 4B).

The evidence that we could find for a recombinant origin of ToLCVs was that the genome region corresponding to the IR or CR is highly divergent relative to the analogous genome region in other geminiviruses, and has potentially been derived through recombination from either a highly divergent geminivirus lineage or another source entirely. From the recombination analysis and phylogenetic results, it is clear that these exhibit multiple putative recombinations between themselves and also unknown viruses. The A components of all the viruses in Asian continent share a common backbone from ToLCV and have integrated other pieces of DNA that have been said to originate from the other viruses not identified so far.

## Discussion

The molecular characterization of viruses with circular genome has become very convenient with the use of the RCA technique, which can detect the presence of DNA A/B genomes and beta satellites without any prior information about the virus sequence. The present study confirmed the presence of representatives of two distinct isolates of ToLCV in India, viz., ToLCV-CTM and ToLCV-K3/K5 that exhibit maximum homology to CrYVMV and ToLCPV isolates, respectively. Determination of the complete DNA-A nucleotide sequences of these isolates suggests that the two isolates of ToLCV from India are about 16% different from each other, while each of them is more than 12% different from most of the begomoviruses [15]. It is also worthwhile to note that in both the cases one of the putative recombination sites is present in the IR regions of the genomes. The crossover during recombination might have occurred close to the conserved stem loop structure of the IR in which the origin of replication (*ori*) resides, as the *ori *has been suggested as the hot spot for recombination in the geminiviruses [16,17]. However, the existence of additional recombinations can not be ruled out [18,19]. For example, the IR region of ToLCV-K3/K5 isolates show highest homology (91%) to Whitefly transmitted Indian begomovirus from *Parthenium hysterop *(DQ339128) and not with ToLCV-BII or ToLCV-RYK. Considering the overall sequence identity of both components and the fact that sequence differences are scattered all along their genome, it is possible that the two viral isolates from North and South India have separated long time ago and are not the result of a recent introduction in either direction. Recombination events have been shown to be key factors in the development and spread of ToLCV infections and it has been suggested that recombination is a significant contributor to geminivirus evolution [20]. Since not much is known about the root of the geminivirus evolutionary tree, it is difficult to determine at this stage which geminivirus genera share more recent common ancestry.

Under field and glasshouse conditions it was observed that ToLCV-CTM DNA A alone was sufficient to establish and spread infection in the tomato plants (Figure 1A). On the other hand, it was noticed that tomato plants cv Pusa Ruby agroinfiltrated with ToLCNDV A and B genomes together leads to leaf mottling and yellowing (Figure 1B). We also observed that the ToLCV-CTM genome infection specifically led to upward leaf curling (as shown in Figure 1A) and is not the result of infection by bipartite geminiviruses. It is likely that ToLCV-CTM A genome (both in field and glasshouse) is able to sustain infection. A number of monopartite begomoviruses have been reported to be associated with betasatellite molecules, which depend on the helper virus for their proliferation, movement and transmission between plants and, in turn, help the virus accumulation and symptom expression [4,21]. In this case, we could not detect the presence of any second genomic component (betasatellite or DNA-B), as revealed by PCR or the RCA by the phi-29 polymerase technique. The experiments with previously characterized genomes of monopartite viruses with no associated betasatellite, indicate that ToLCV genomes are capable of replication, symptom development and induction of plant defence response even in the absence of coat protein [18] and pre-coat protein [22]. This study reports the presence of new strains of geminiviruses that infect tomato plants, which have single genomic component. Clearly, the transcription of this single component produces all the gene products required to support a complete cycle of infection and transmission of the virus.

## Conclusion

We have characterized two new *Tomato leaf curl viruses *(ToLCV-CTM and ToLCVK3/K5) with single genomic component and demonstrated the association of these isolates with the symptomatic tomato leaf curl samples obtained from two geographically distant niches of Indian subcontinent. Through the evaluation of the genetic variation of these ToLCV isolates from India, we found that there is uneven nucleotide variation along the genome and the region corresponding to the IRs is highly divergent relative to the analogous genome region in other geminiviruses. It is clear from the sequence analysis and phylogenetic results that the genomes of these isolates exhibit multiple putative recombination events between themselves and have integrated other pieces of DNA that have been presumably originated from the other viruses not identified so far. This study also demonstrates the wide sequence variability amongst ToLCV isolates from India. There is an urgent need for more information in order to develop effective and sustainable approaches to manage the diseases caused by these plant pathogens.

## Methods

### Provenance of the virus material and genomic DNA extraction

More than 80% of the tomato plants in the fields showed severe ToLCV symptoms with tomato in the ToLCV-CTM samples obtained from the central region of New Delhi, India in the middle of February 2006 and from the fields of Kerala (a southern state of India) in April 2008. Due to the association of whiteflies with the plants, begomovirus infection was suspected to cause the disease in the samples. Tomato plants exhibiting stunted growth, severe leaf curling, reduced leaf size and distortion of leaf lamina symptoms associated with ToLCV infection were selected for sampling. Symptoms of infected tomato samples collected in the field were reproduced under glasshouse conditions to examine symptom variability. Three weeks old healthy tomato seedlings were grown along with the infected tomato plants harboring whiteflies that were chosen for sampling. In all the cases, the symptoms expressed in the field were reproduced in the growth chamber and plants failed to recover from the disease even after 6 months of infection. Total DNA from infected leaves was extracted following the standard CTAB protocol [23].

### Rolling circle amplification and cloning of full length viral genome

The coat protein fragment was detected from infected, symptomatic samples using the BGCPF (5'-TGTGARGGYCCWTGYAARGTYCA-3') and BGCPR (5'-TASARGCATGWGTACANGCCATATAC-3') primers. The rolling circle amplification (RCA) was performed for the samples ToLCV-K3/K5 and ToLCV-CTM using the TempliPhi 100 Amplification kit (Amersham Biosciences, USA) following the manufacturer's instruction. The RCA products were digested with *BamH *I enzyme (New England Biolabs) for ToLCV-K3/K5 and ToLCV-CTM respectively to get a band of an approximate 2.7 kb size for cloning into a suitable vector. The digested products were checked on 1.2% agarose gel run at 60 Volts to separate the DNA A and DNA B genomes (if present). These were cloned in cloning vector pCAMBIA1391Z and sequenced.

### Identification of genes, Sequence analysis and phylogenetic study

Three replicate clones from the PCR products were sequenced to minimize any error. Sequence data of all the components obtained were compared with other reported isolates of *Tomato leaf curl virus *from Indian subcontinent as well as with those reported from other parts of the world. All the open reading frames (ORFs) that could potentially express proteins were used in protein-protein BLAST (BLASTp) [24] searches to identify potential homologues of these in the NCBI non-redundant protein sequences database and a nested search strategy to restrict the search to geminivirus protein sequences. Nucleotide/amino acid sequence similarities were carried out with isolates reported for each component from various begomovirus isolates (Table 1) using Clustal W (Version 1.82) [25] and Bio-Edit (Version 7.0.0) softwares. The phylogenetic trees were constructed from the multiple alignments by the neighbour-joining majority rule consensus. The degrees of similarity shared by full-length genome sequences of various representative geminiviruses (see Table 1) were graphically depicted using a neighbour joining tree (1000 bootstrap replicates). Using RDP3 software http://darwin.uvigo.es/rdp/rdp.html, we performed analysis of various DNA-A sequences for the identification of potential recombinants, parental sequences, and approximation of possible recombination breakpoint positions. The pairwise comparison of sequences was carried out between sequences of different species and of different isolates and an average profile for the considered cluster of viruses was calculated for these two categories taking 100 nt along the genome sequence. The analyses were performed with default settings for the detection methods, a Bonferroni-corrected p-value cutoff of 0.05. The approximate breakpoint positions and recombinant sequence(s) inferred for every detected potential recombination event were manually checked and adjusted where necessary using the extensive phylogenetic and recombination signal analysis features available in RDP3. Once a set of unique recombination events was identified, a breakpoint map containing the positions of all clearly identifiable breakpoints was compiled. The sequences used for the analysis include EU910140, AJ507777, U38239, DQ116884 and DQ852623.

## Competing interests

The authors declare that they have no competing interests.

## Authors' contributions

PP, SKM, and GSS designed and conceptualized the study, PP and SM executed the experiments, PP and ARN carried out the sequence analysis, alignment and Phylogenetic studies, and PP, ARN and NRC prepared the manuscript. All authors read and approved the final manuscript.

## Supplementary Material

Additional File 1 **Table S1**. There is one supplemental table which includes the RDP3 analysis. **Table S1: **The summary of the RDP3 analysis for possible recombination events among the viral isolates identified in this study and those exhibiting close homology. Click here for file
