# Supplementary material for: Molecular characterization of two distinct monopartite begomoviruses infecting tomato in india
Source: Virol J. 2010 Nov 23;7:337. doi: 10.1186/1743-422X-7-337 (PMC3002923; doi:10.1186/1743-422X-7-337)
Supplement: Additional File 1 — Table S1. There is one supplemental table which includes the RDP3 analysis. Table S1: The summary of the RDP3 analysis for possible recombination events among the viral isolates identified in this study and those exhibiting close homology. [file 1743-422X-7-337-S1.DOC]

**Supplementary Table S1. Recombination analysis of DQ629102**

| **Sequence Origin** | **Beginning breakpoint** | **Ending breakpoint** | **Major parent** | **Minor parent** | **Probability** |
| --- | --- | --- | --- | --- | --- |
| DQ852623 | 1372 | 1455 | EU910140 | DQ852623 | 4.636X10^-04^ |
| U38239 | 2058 | 2179 | EU910140 | U38239 | 5.376X10^-04^ |
| U38239 | 2554 | 2602 | EU910140 | U38239 | 2.004X10^-03^ |

**Supplementary Table S2. Recombination analysis of K3/K5 (EU910141/ EU910140)**

| **Sequence Origin** | | **Beginning breakpoint** | **Ending breakpoint** | **Major parent** | **Minor parent** | **Probability** |
| --- | --- | --- | --- | --- | --- | --- |
| **K3** | DQ852623 | 2434 | 2510 | AJ507777 | DQ852623 | 3.275X10^-05^ |
|  | U38239 | 2308 | 2326 | AJ507777 | U38239 | 8.814 X10^-04^ |
| **K5** | U38239 | 2498 | 2508 | DQ116884 | U38239 | 1.387 X10^-02^ |
|  | DQ116884 | 24 | 982 | U38239 | DQ116884 | 3.26 X10^-74^ |
